# Human immunodeficiency virus and type 2 diabetes

**DOI:** 10.1080/17571472.2017.1302872

**Published:** 2017-03-19

**Authors:** P. Avari, S. Devendra

**Affiliations:** a Specialist Training Registrar in Endocrinology, London Deanery (North West Thames), London, UK; b Acute Medicine & Endocrinology, West Hertfordshire Hospitals NHS Trust, Watford, UK

**Keywords:** Human immunodeficiency virus, HIV, diabetes, HAART, antiretroviral therapy

## Abstract

The prevalence of diabetes is higher amongst individuals infected with HIV. The major contributor to hyperglycaemia is thought to be iatrogenic, with protease inhibitors being most commonly associated to insulin resistance. This article is to update general practitioners on the diagnosis and management of diabetes in HIV-infected patients. Specific considerations are highlighted including interactions of particular diabetic drugs with antiretroviral therapy (ART). We articulate why the use of Hemoglobin A1c (HbA1c) testing is not recommended as a diagnostic tool.

**Case history**

A 48 year old businessman with an 8 year history of being HIV positive was notified recently that he has type 2 diabetes mellitus. His recent HbA1c was 8.4% DCCT (68 mmol/mol IFCC). His BMI is currently 30 kg/m2. He has no microvascular or macrovascular complications from diabetes. He has an erratic work and eating pattern. Although he has seen a dietician in the past, he does not adhere to a strict healthy diet. From his HIV point of view, he has been on anti-retroviral therapy (ritonavir/atazanir/tenofovir). However his main complaint is erectile dysfunction.

**Why this matters to me?**

Co-existence of diabetes to HIV infection adds complexity to the standards of care. The aim of this article is to update physicians on the diagnosis and management of type 2 diabetes mellitus (T2DM) in HIV-infected patients and highlight the specific considerations with respect to diagnosis, use of Haemoglobin A1c (HbA1c) testing and the interactions of particular antidiabetic drugs with antiretroviral therapy (ART).

## Key messages

(1) Diabetes is four times more common in HIV-infected men exposed to anti retroviral therapy than in HIV seronegative men.
(2) Protease inhibitors based antiretroviral treatment is most commonly associated with increased incidence of diabetes.
(3) HbA1c is not recommended for screening for diabetes in HIV infected individuals.
(4) Fasting serum glucose and lipid levels should be measured in HIV-positive patients before beginning antiretroviral therapy and used for diagnosis. Oral glucose tolerance test may be considered in those with high risk.
(5) Consult with specialist if considering switching anti-retrovirals

## 1. Is there is a link between type 2 diabetes mellitus and HIV?

A recent study has shown that diabetes is up to four fold more common in HIV-infected men exposed to Highly Active Anti-Retroviral Therapy (HAART) than in HIV seronegative men [1]. The exact mechanism for the development of insulin resistance is yet to be defined, but several risk factors for the development of diabetes in HIV-affected individuals have been identified as outlined in Table 1.

**Table 1.** Aetiology of type 2 diabetes mellitus in HIV infected individuals.

Diabetes due to HIV disease	Diabetes as a result of iatrogenic factors
• Endocrine abnormalities (e.g. growth hormone deficiency in HIV contributes to insulin resistance)	• Exposure to Anti Retroviral therapy: • causes insulin resistance • decreases insulin secretion • Fat distribution changes leading to lipodystrophy • B-cell toxicity
• Hepatitis C infection	
• Viral factors: viral burden, lower CD4 count, duration of viral infection	
• Inflammation caused by HIV	

Source: Adapted from Kalra et al. [3].

Whilst over the past few decades HAART has significantly improved the clinical outcomes of HIV patients, HAART has also led to an increase in metabolic dysfunction. The HIV lipodystrophy syndrome is characterised by insulin resistance, an abnormal lipid profile and peripheral fat wasting with central fat (visceral) accumulation. Increased visceral fat with wasting of subcutaneous fat, creates higher levels of inflammatory cytokines such as TNFa. This leads to diabetes or impaired glucose tolerance by increasing insulin resistance [2].

## 2. How frequently should an individual with HIV be screened for diabetes?

There are no UK specific guidelines to guide us on this subject, however the latest American Diabetes Association has published guidelines for the diagnosis and management of diabetes in HIV infected people which are summarised in Table 2. Whilst these guidelines advise fasting blood glucose as a screening tool, the predominant role of insulin resistance implies that post-prandial glucose values, or an oral glucose tolerance test, may also be performed as part of screening measures [4].

**Table 2.** American Diabetes Association 2016 guidelines for the diagnosis and management of diabetes in HIV infected people [5].

• Fasting glucose levels and lipid levels (such as cholesterol and triglycerides) should be measured in HIV-positive patients before beginning antiretroviral therapy
• Screen for diabetes 3 months after initiating therapy or changing HAART, and then annually afterwards
• Where pre-diabetes is identified, measure glucose levels every 3–6 months
• HbA1c levels are not recommended for use as a diagnostic tool in HIV-positive patients (please see Section 4)

## 3. Can HAART contribute to his development of diabetes?

The major contributor to hyperglycaemia in HIV/AIDS is actually thought to be iatrogenic. Certain classes of antiretrovirals have been shown to increase the likelihood of developing diabetes in HIV infected subjects, such as protease inhibitors (PIs). PIs interfere with GLUT-4-mediated glucose transport, which is the rate limiting step in glucose uptake in to muscle and adipose tissue, resulting in increased insulin resistance [6]. Risk factors for development of diabetes with PI therapy include positive family history of diabetes, weight gain, lipodystrophy, old age and hepatitis C infection.

All PIs do not have the same metabolic effects. Whilst indinavir induces insulin resistance with no effect on lipid metabolism, iopinavir and ritonavir increase fasting triglycerides and free fatty acids, but do not worsen insulin sensitivity. Indinavir and ritonavir both block GLUT-4, but no such effect is noted with amprenavir, and atazanzvir.

The other class of drugs which is used is the nucleoside analogs (NRTIs). It was earlier felt that NRTIs were less likely to cause metabolic abnormalities; however, it has been shown that these drugs, too, increase the risk of diabetes. The risk is highest with stavudine, but is also significant with zidovudine and didanosine. Proposed mechanisms include insulin resistance, lipodystrophy and mitochondrial dysfunction. These mechanisms may be evident only in HIV-infected persons treated for long periods of time with NRTIs [7].

Despite the association between HAART and the development of diabetes, this does not mean that HAART should not be prescribed to patients with HIV and diabetes. Physicians need to be aware of the predominant mechanism of diabetes linked to each drug, so that appropriate anti-diabetic therapy can be chosen.

Currently, there is no evidence that non-nucleoside reverse transcriptase inhibitors, integrase inhibitors and CCR5 antagonists increase the risk for diabetes in people with HIV (Table 3).

**Table 3.** The impact of the main HIV medications on glucose homeostasis.

Class of drugs	Effect on glucose homeostasis	Mechanism
Protease Inhibitors (PIs)	Yes	Indinavir + ritovir block GLUT4 causing insulin resistance Amprenavir & atazanazvir have no effect on this mechanism Indinavir increases hepatic glucose production and release
Nucleoside Reverse Transcriptase Inhibitors (NRTIs)	Yes	Stavudine increases insulin resistance and lipodystrophy Stavudine, zidovudine and didanosine are associated with increased lactate levels
Non-nucleoside Reverse Transcriptase Inhibitors (NNRTIs)	No	

Source: Adapted from Kalra et al. [8].

## 4. Why is HbA1C testing *not recommended* as a diagnostic tool for diabetes in HIV-positive patients?

HbA1c is the percentage of glycated haemoglobin and is a reflection of long-term glucose status. The HbA1c may underestimate glycaemia in HIV-positive individuals. Greater HbA1c-glucose discordance has been shown in patients with higher mean corpuscular volume (MCV), concurrent NRTI use (esp. abacavir) and lower CD4 count. This is may be due to HIV infected individuals having a faster turnover of red blood cells. In view of the possible discordance between HbA1c and glycaemic control, it is felt a fasting blood sugar is prudent for the diagnosis of diabetes [9].

## 5. What is the treatment of diabetes in HIV patients? Are there any interactions between antidiabetic drugs and antiretrovirals?

There are very few clinical trials to suggest that the treatment of diabetes is any different from that in the non-HIV population. A multifactorial approach is pertinent and includes not only glucose control, but also blood pressure and dyslipidaemia management, as well as advice to stop smoking and increase physical activity.

Whilst there is no specific evidence base, the relative advantages and disadvantages of some therapies over others need to be taken into account (Table 4).

**Table 4.** Oral diabetic medications with special considerations in HIV.

Name	Mechanism	Special considerations in HIV
Biguanide (Metformin)	• First line drug of choice • Improves insulin sensitivity • Decreases hepatic glucose concentration	• Dolutegravir increases metformin concentration therefore may require reduction in dose • Lactic acidosis can be caused by certain NRTIs (e.g. stavudine)
Sulphonylureas	• Stimulates insulin release from pancreatic B cells • Reduced glucose output from the liver • Increases insulin sensitivity	• Risk of hypoglycaemia • Particularly useful for patients aiming to gain weight
Thiazolidinediones (glitazones)		• Contraindicated in hepatic dysfunction and heart failure • When used with CYP2B inhibitors (many PIs), rosiglitazone/ pioglitazone levels may increase. Need to monitor carefully
Gliptins (DDP-4 inhibitors)	• Increases incretin levels (GLP-1 and GIP), which inhibits glucagon release • Increases insulin secretion and reduces gastric emptying	• Saxagliptin interacts with CYP3A4 inhibitors (e.g. ritonavir); therefore avoid saxagliptin or prescribe at a lower dose
SGLT-2 inhibitors	• Reduce reabsorption of glucose • Increases urinary excretion of glucose	• If canagloflozacin is co-administered with UDP-gluconosyltransferase enzyme inducers (e.g. ritonavir), consider increasing dose to 300 mg
GLP-1 analogues	• Increases glucose-dependent insulin secretion • Decreases inappropriate glucagon secretion • Slows gastric emptying	

Source: Adapted from Monroe et al. [12].

### 5.1. Metformin

Metformin is the first line drug of choice in most individuals with T2DM, but should be used with caution in HIV. Weight loss caused by metformin can often worsen lipoatrophic areas, resulting in a deterioration of various metabolic parameters. Lactic acidosis can be caused by certain NRTIs (e.g. stavudine) and thereby be potentiated by metformin. The HIV integrase inhibitor dolutegravir increases plasma exposure and may require lowering of metformin dose.

### 5.2. Sulphonylureas

In HIV lipodystrophy syndrome, insulin resistance appears to be the predominant pathophysiology rather than insulin secretion. Sulphonylureas stimulate the pancreas to produce insulin, and additionally can lead to hypoglycaemia. However, they remain useful adjuncts to insulin sensitisers particularly for patients aiming to gain weight [10].

### 5.3. Thiazolidinediones (glitazones)

These have a mechanism of action which should make them drugs of choice in HIV. The possibility of slight increase in subcutaneous fat makes them the preferred drug class in patients with lipodystrophy. However, these drugs are contraindicated in hepatic dysfunction and heart failure. There is also an increased risk of bladder cancer with pioglitazone. They may cause oedema, increase cardiovascular morbidity, worsen osteoporosis and hence these side effects have prevented wide usage of these drugs in individuals with T2DM, as well as HIV-associated diabetes.

### 5.4. Gliptins (DDP-4 inhibitors)

There are limited studies examining the effect of DDP-4 inhibitors with antiretrovirals, however, saxagliptin interacts strongly with CYP3A4 inhibitors, such as ritonavir, so should be avoided or be prescribed at a reduced dose [11].

### 5.5. SGLT-2 inhibitors

SGLT-2 inhibitors, such as dapagliflozin and canagliflozin block reabsorption of glucose in the proximal tubule, and result in glucose excretion. The advantages include weight loss (approximately 2 kg), lower BP and reduced risk of hypoglycaemia. The main disadvantages include glycosuria resulting in urinary tract and genital fungal infections. No interaction between ART and dapagliflozin has been noted, but if canagliflozin is co-administered with UDP-gluconosyltransferase enzyme inducers such as ritonavir, clinicians should consider increasing the dose to 300 mg [12].

### 5.6. GLP-1 analogues

Incretin mimetics are likely to have similar effects to those seen in the general population, as those in HIV-infected individuals. Liraglutide has been reported to improve various parameters including insulin sensitivity, blood pressure and weight, apart from achieving effective hypoglycaemic control [13]. Further studies are underway to study the effect of this class of antihyperglycaemics in HIV-associated diabetes.

### 5.7. Insulin

Insulin therapy is required in patients where glycaemic control cannot be achieved with oral treatments alone. Insulin has the theoretical benefit being an anabolic hormone of weight gain which may be particularly advantageous in HIV positive individuals with diabetes with low BMI. It is recommended as first-line therapy for patients with HbA1c DCTT >9% (or >75 mmol/mol IFCC), severe liver or kidney disease. The choice of insulin is dependent on the clinical needs and patent lifestyle – as with non-HIV patients.

### 5.8. Switching antiretroviral regimes

Switching antiretroviral regimes may be considered if a patient is on lopinavir/ritonavir or a thymidine analogue (stavudine, zidovudine), however there is uncertain benefit in switching other medications. The alternate regime should be less metabolically disruptive, but equally efficacious in managing HIV. The decision to switch antiretrovirals should be made by an HIV specialist.

Protease Inhibitors based HAART should ideally be avoided in patients with a high risk of developing diabetes e.g. previously having gestational diabetes, a positive family history, or impaired glucose tolerance on testing [2].

## 6. Is HIV associated with low testosterone? How would you manage his erectile dysfunction?

HIV-infected men have a higher prevalence of erectile dysfunction (ED) than age-matched controls. The prevalence of ED has been noted to be as high as 70% in out-patient studies. Erectile dysfunction can be related to factors other than the HIV infection per se such as age, smoking, alcohol consumption, testosterone levels, prolactin levels, stress/psychological factors, other medications including antidepressants, steroids and anti-hypertensives.

History and medical examination is prudent to exclude hypogonadism. Laboratory investigations include testosterone (to evaluate for hypogonadism – check in the morning for greatest accuracy), FSH, LH, prolactin, fasting glucose, HbA1c, lipids and TSH.

## 7. Outcome of the case

Our patient was referred to a diabetologist and was managed in conjunction with an HIV specialist. A decision was made to continue on the current antiretroviral regime and the patient was started on metformin. He was referred to a dietician and advised to lose weight and increase his physical activity. He was investigated for his erectile dysfunction, and found to have primary hypogonadism, for which he was started on testosterone replacement (nebido injections).

## Disclosure statement

No potential conflict of interest was reported by the authors.
